# Post-ischemic inhibition of the mitochondrial Ca^2+^ uniporter with Ru265 protects in *ex vivo* and *in vivo* models of cerebral ischemia

**DOI:** 10.1042/CS20261842

**Published:** 2026-10-15

**Authors:** Yuliya V. Medvedeva, Hong Z. Yin, Afsheen Bazrafkan, Masih A. Rafi, Evelyn Wang, Ivan A. Yeromin, Edward Sharman, Humza Ghaznavi, Sepehr Khatami, Kantapon Suayngam, Alexander L. Liu, Najwa W. Ellahib, Renee E. Lotfy, Justin J. Wilson, Yama Akbari, John H. Weiss

**Affiliations:** 1 Department of Neurology, University of California Irvine, Irvine, CA 92697, United States; 2 Department of Anatomy & Neurobiology, University of California Irvine, Irvine, CA 92697, United States; 3 Department of Neurological Surgery, University of California Irvine, Irvine, CA 92697, United States; 4 The Beckman Laser Institute & Medical Clinic, University of California Irvine, Irvine, CA 92697, United States; 5 Department of Chemistry and Biochemistry, University of California, Santa Barbara, 93106, Santa Barbara, CA, United States

**Keywords:** brain ischemia, cardiac arrest, hippocampal slice, MCU, mitochondria, zinc

## Abstract

Brain ischemia is a leading cause of morbidity and mortality in the aging population, yet no effective neuroprotective interventions currently exist. This therapeutic gap stems from two key challenges: the inability to deliver neuroprotective drugs before reperfusion and limited understanding of post-reperfusion events leading to delayed neurodegeneration. Zn^2+^ is an important contributor to ischemic brain injury, with mitochondrial Zn^2+^ uptake playing a central role in its neurotoxic effects. Ongoing Zn^2+^ accumulation occurs through the mitochondrial Ca^2+^ uniporter (MCU) in selectively vulnerable CA1 neurons after transient ischemia, contributing to delayed mitochondrial dysfunction. We previously demonstrated that post-ischemic administration of the non-selective MCU antagonist, ruthenium red attenuated neuronal damage in both an *ex vivo* oxygen–glucose deprivation (OGD) mouse brain slice model and in an *in vivo* rat cardiac arrest (CA) model. In the present study, we test post-ischemic treatment with Ru265, a novel selective MCU blocker, in both ischemic models. In the slice model, OGD induced delayed Zn^2+^ accumulation in CA1 neuronal mitochondria (as previously reported), followed by late-onset persistent mitochondrial depolarization and sustained elevation of cytoplasmic Ca^2+^ levels, indicating loss of Ca^2+^ homeostasis. Application of Ru265 after OGD termination inhibited the mitochondrial Zn^2+^ accumulation and largely prevented subsequent mitochondrial depolarization and loss of cytosolic Ca^2+^ homeostasis. In the CA model, intravenous Ru265 administration during cardiopulmonary resuscitation largely mitigated mitochondrial damage, markedly reduced neuronal injury and improved behavioral outcomes 20–24 h after ischemia. These findings suggest that post-ischemic MCU blockade may constitute a promising neuroprotective therapeutic strategy.

## Introduction

Brain ischemia remains a leading cause of morbidity and mortality, particularly among the aging population. Despite decades of research, effective neuroprotective interventions have yet to be established in clinical practice [1]. To date, cardiopulmonary resuscitation (CPR) represents the only clinically proven treatment for improving neurological outcomes following cardiac arrest (CA). Although therapeutic hypothermia after resuscitation offered hope in early clinical studies, recent trials have questioned its benefits [2]. Similarly, prompt restoration of cerebral blood flow remains the sole evidence-based intervention for ischemic stroke. However, recent advances in cerebral revascularization techniques following stroke [3] have extended the therapeutic window and created new opportunities for adjunctive neuroprotective therapies.

Until recently, most treatments demonstrated to be effective in animal models were administered before the onset of ischemia. In clinical settings, however, opportunities for intervention typically arise only after the restoration of blood flow. Notably, following brief ischemia, select neuronal populations—particularly those in the hippocampal CA1 region and cortical layers 3, 5, and 6—initially survive but subsequently undergo delayed neuronal death over the ensuing hours to days, a phenomenon well-documented in both rodents and humans [4–9]. These selectively vulnerable neurons represent potential targets for therapeutic salvage with timely interventions. Therefore, elucidating the sequence of post-ischemic molecular and cellular events that drive neurodegeneration in these vulnerable populations is essential for developing effective neuroprotective strategies.

Mitochondria have been consistently identified as primary targets of ischemic injury. Transient ischemia induces early mitochondrial damage and dysfunction, culminating in ATP depletion, reactive oxygen species (ROS) overproduction, and the release of pro-apoptotic mediators [10–12].

Accumulating evidence has implicated Zn^2+^–mitochondria interactions as a key contributor to ischemic neuronal injury [13,14]. Consistent with this, we recently observed prolonged Zn^2+^ accumulation within mitochondria of selectively vulnerable CA1 neurons—but not in the comparatively resistant CA3 neurons—occurring after transient ischemia [15]. Notably, delayed administration of either the Zn^2+^ chelator N,N,N’,N’-tetrakis(2-pyridylmethyl)ethylenediamine (TPEN) or ruthenium red (RR), an antagonist of the mitochondrial Ca^2+^ uniporter (MCU; the major route for Zn^2+^ entry to mitochondria), attenuated both mitochondrial and neuronal damage at 4 h post-ischemia in the *ex vivo* acute hippocampal slice model. RR was further shown to confer neuroprotection in an *in vivo* rat CA model [16]. However, RR is highly non-selective, exerting numerous off-target effects beyond MCU blockade, and is unsuitable for human use [17]. Likewise, the alternative MCU inhibitor RU360 presents significant translational limitations, as it is membrane-impermeable and susceptible to redox transformations that render it inactive [18].

The present study investigated the neuroprotective potential of Ru265 [19], a recently synthesized MCU inhibitor that is substantially more potent, selective, and cell-permeable than previously available MCU blockers, offering considerable translational potential. Acute hippocampal slices were subjected to transient oxygen–glucose deprivation (OGD, 8 min) followed by reperfusion to model ischemia–reperfusion injury. Brief OGD induced persistent Zn^2+^ accumulation in CA1 neuronal mitochondria occurring after reperfusion, consistent with our previous observations [15], along with delayed injury at 4 h post-reperfusion, including persistent mitochondrial depolarization and sustained elevation of cytoplasmic [Ca^2+^], indicative of disrupted Ca^2+^ homeostasis.

Delayed post-OGD application of Ru265 suppressed late mitochondrial Zn^2+^ accumulation and largely prevented both mitochondrial depolarization and loss of cytosolic Ca^2+^ homeostasis. In an *in vivo* rat CA model of transient global brain ischemia, intravenous Ru265 administration during CPR markedly attenuated ischemia-evoked mitochondrial swelling and fragmentation, diminished neuronal injury, and improved behavioral outcome 20–24 h post-ischemia.

Taken together, these findings provide compelling support for delayed administration of MCU antagonists as a therapeutic strategy to mitigate post-ischemic injury. Critically, such treatment can be administered following restoration of blood flow—a clinically relevant therapeutic window.

## Materials and methods

### Reagents

Ru265 is a novel, potent (IC50 2.6 nM), and membrane–permeable inhibitor of the MCU. Ru265 was synthesized as previously described [19,20] by the laboratory of Justin Wilson. Ketamine was obtained from Sigma (St. Louis, MO). TOM20 antibodies were obtained from Santa Cruz Biotechnology (Santa Cruz, CA) and DyLight™ 488 antibodies were obtained from Jackson ImmunoResearch (West Grove, PA). All other reagents were purchased from Thermo Fisher Scientific (Waltham, MA).

### Ethics statement

This study was carried out in accordance with the Guide for the Care and Use of Laboratory Animals of the National Institutes of Health and was approved by the Institutional Animal Care and Use Committee (IACUC) at the University of California, Irvine.

### Animals

Efforts were made to minimize animal numbers and suffering. Both male and female mice from 129S6/SvEvTac strain (Taconic Biosciences; 4–5 weeks old) were utilized for *ex vivo* experiments. All *in vivo* experiments were performed using adult male Wistar rats (Envigo, Philadelphia, PA), aged an average of approximately 66 days and weighing 363.35 ± 42.43 g. Rats were housed under standard laboratory conditions (23 ± 2°C, 60–70% humidity, 12 h light/dark cycle) with ad libitum access to food and water. Animals were handled daily for two weeks prior to surgery and were calorically restricted to 25% of normal food intake for 14 h prior to the CA procedure, as standard with our prior publications [21,22].

### Preparation of acute brain slices

Mice were deeply anesthetized with isoflurane, decapitated, and brains quickly removed and placed in ice-cold solution containing (in mM): 220 sucrose, 3 KCl, 1.25 NaH_2_PO_4_, 6 MgSO_4_, 26 NaHCO_3_, 0.2 CaCl_2_, 10 glucose, and 0.42 ketamine (pH 7.35, 310 mOsm, equilibrated with 95% O_2_/5% CO_2_). Coronal brain slices (300 μm) were prepared as previously described [23] using a Leica VT1200 vibratome (Leica, Germany). After preparation, slices were incubated in artificial cerebrospinal fluid (ACSF) containing (in mM): 126 NaCl, 3 KCl, 1.25 NaH_2_PO_4_, 1 MgSO_4_, 26 NaHCO_3_, 2 CaCl_2_, 10 glucose (pH 7.35, 310 mOsm, equilibrated with 95% O_2_/5% CO_2_) for 1 h at 34 ± 0.5°C, and then kept at room temperature (20–23°C) in oxygenated ACSF. All experiments were performed at 32 ± 0.5°C.

### OGD/reperfusion in slices and Ru265 administration

To induce short hypoxia-hypoglycemia, ACSF was changed to identical solution without glucose and equilibrated with 95% N_2_/5% CO_2_ (see Figure 1A for experimental timeline). After 8 min, OGD was terminated by restoration of oxygenated ACSF containing glucose. The duration of OGD was determined by our previous experiments with the aim to withdraw OGD before Ca^2+^ deregulation occurs in the majority of CA1 neurons, while allowing sufficient time to evoke mitochondrial depolarization (loss of ∆Ψ_m_), Zn^2+^ accumulation and delayed (4 h later) mitochondrial dysfunction [15,23].

**Figure 1:**
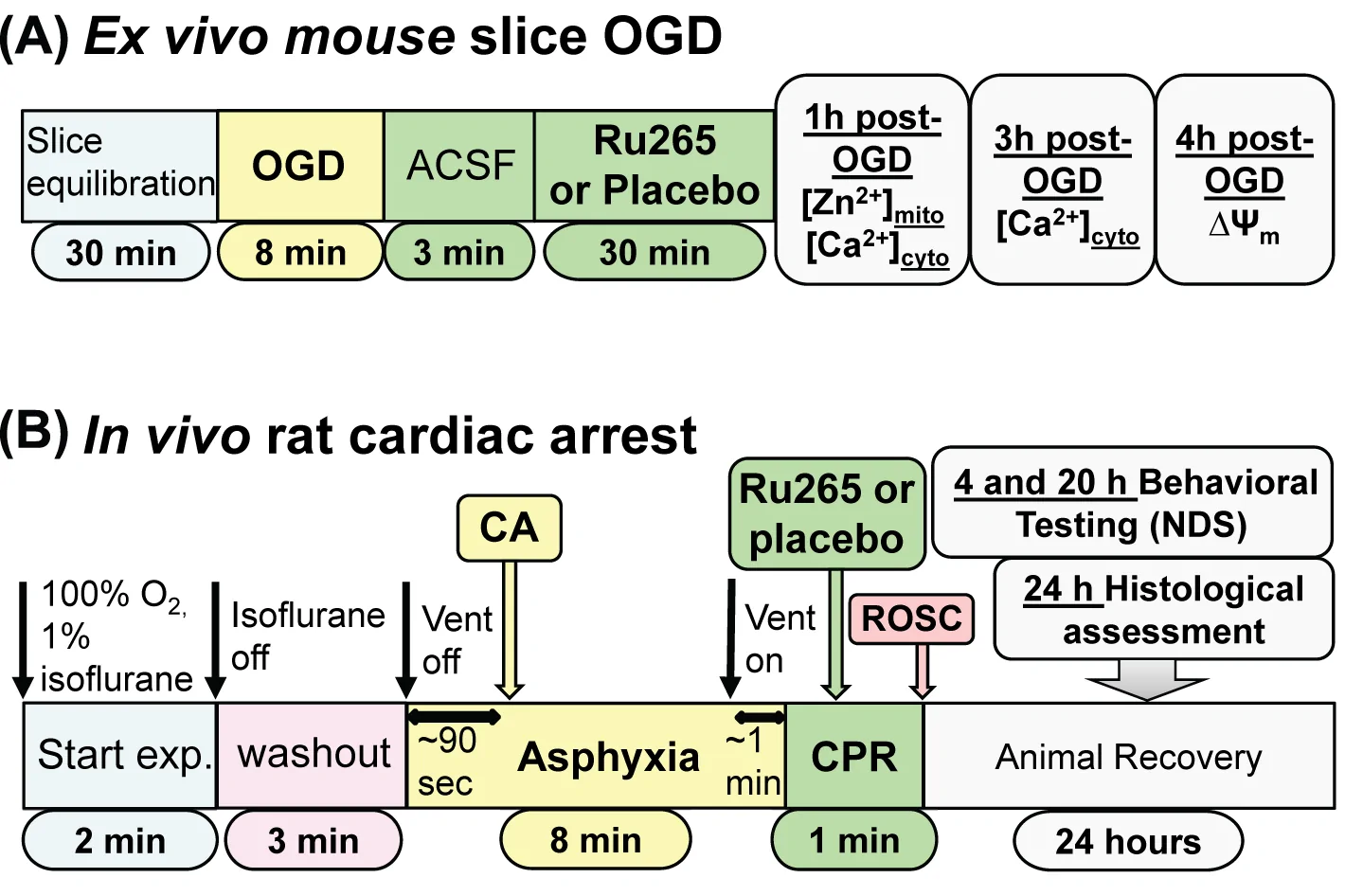
Experimental procedure (**A**) OGD in brain slices. Schematic show steps of experiment modeling ischemia/reperfusion in acute brain slices. Experiment was terminated at 4 h. Intramitochondrial Zn^2+^ levels were assessed at 1 h, and cytoplasmic Ca^2+^ levels were evaluated at 1 and 3 h after OGD withdrawal. (**B**) *In vivo* CA experimental procedure. Schematic represents steps of an experiment modeling CA evoked transient global ischemia in rats. Ru265 was administered at the time of CPR along with standard intravenous epinephrine. All animals were intubated, attached to ventilator, and cannulated before CA or sham procedure. Chest compressions were performed on sham animals to mimic CPR. Behavioral testing was performed at 4 and 20 h after CPR. At 24 h experiment was terminated, rats euthanized and brains processed for histological assessments.

Ru265 (5 μM) was applied 3 min after the OGD termination for 30 min.

### Fluorescent measurements

#### Measurements of intracellular Zn^2+^ in individual hippocampal neurons

Slices were placed in a flow-through chamber (RC-27L, Warner Instruments) mounted on the stage of an upright microscope (BX51WI Olympus, Japan) and perfused with oxygenated ACSF at 2 ml/min. Membrane-impermeable Zn^2+^ indicator FluoZin-3 [24,25] and Ca^2+^ indicator (either low affinity Fura-FF or high affinity Fura-2) were dissolved in pipette solution (containing, in mM: 125 K-gluconate, 10 KCl, 3 Mg-ATP, 1 MgCl_2_, 10 HEPES, pH 7.25 with KOH, 290 mOsm with sucrose) at a concentration of 1 mM, and 1 μl placed in the tip of a patch-pipette (5–7 MΩ, borosilicate glass with filament), prior to backfilling with pipette solution. Neurons were loaded with indicator via patch-pipette which was slowly withdrawn after ∼5 min, as previously described [23]. The intracellular Ca^2+^ level (as Fura-FF or Fura-2 ratio) was monitored during pipette withdrawal. If Fura ratio increased during withdrawal, even briefly, the cell was considered damaged and was not used. Fluorescence was excited at 340(20), 380(20), and 482(20) nm via a ×40 water-immersion objective, using a Xenon light source (Sutter Instruments), and emitted fluorescence collected at 532 (40) nm using a cooled CCD camera (Hamamatsu, Japan). All filters are bandpass with bandwidths indicated in parentheses. Images were acquired every 15 or 30 s. After background subtraction, images were analyzed using METAFLUOR 7.1 software (Universal Imaging). FluoZin-3 fluorescence changes are presented as δ*F*/*F*_0_ = (*F* − *F*_0_)/*F*_0_ × 100%, where *F* is the current fluorescence intensity, and *F*_0_ is the average baseline fluorescence during the 10 min prior to OGD. The photobleaching-related decrease in FluoZin-3 fluorescence averaged 4–7% per 10 min, depending on exposure time. If the rate of fluorescence decrease accelerated considerably during the experiment (>10%) the cell was withdrawn from the experiment. For background subtraction, we determined a fluorescence-free region for each recording located at least 100 μm from the loaded neuron(s). 1–2 neurons per slice were loaded, and only neurons that remained in sharp focus throughout the whole experiment were used.

To assess the level of mitochondrial Zn^2+^ accumulation following OGD, a high dose of carbonyl cyanide-4-(trifluoromethoxy)phenylhydrazone (FCCP, 2 μM, 5 min) was administered to the slice ∼1 h after OGD withdrawal. This treatment causes dissipation of the mitochondrial potential (∆Ψ_m_) and release of ions accumulated within mitochondria to the cytoplasm, where they can be sensed by cytoplasmic fluorescent indicators. We assessed two parameters: the maximum amplitude of FluoZin-3 fluorescence increase within 15 min after the start of the FCCP application (see Figure 2B); and the area under the FluoZin-3 curve, evaluated for 15 min starting from the onset of the of FCCP exposure (Figure 2B).

**Figure 2:**
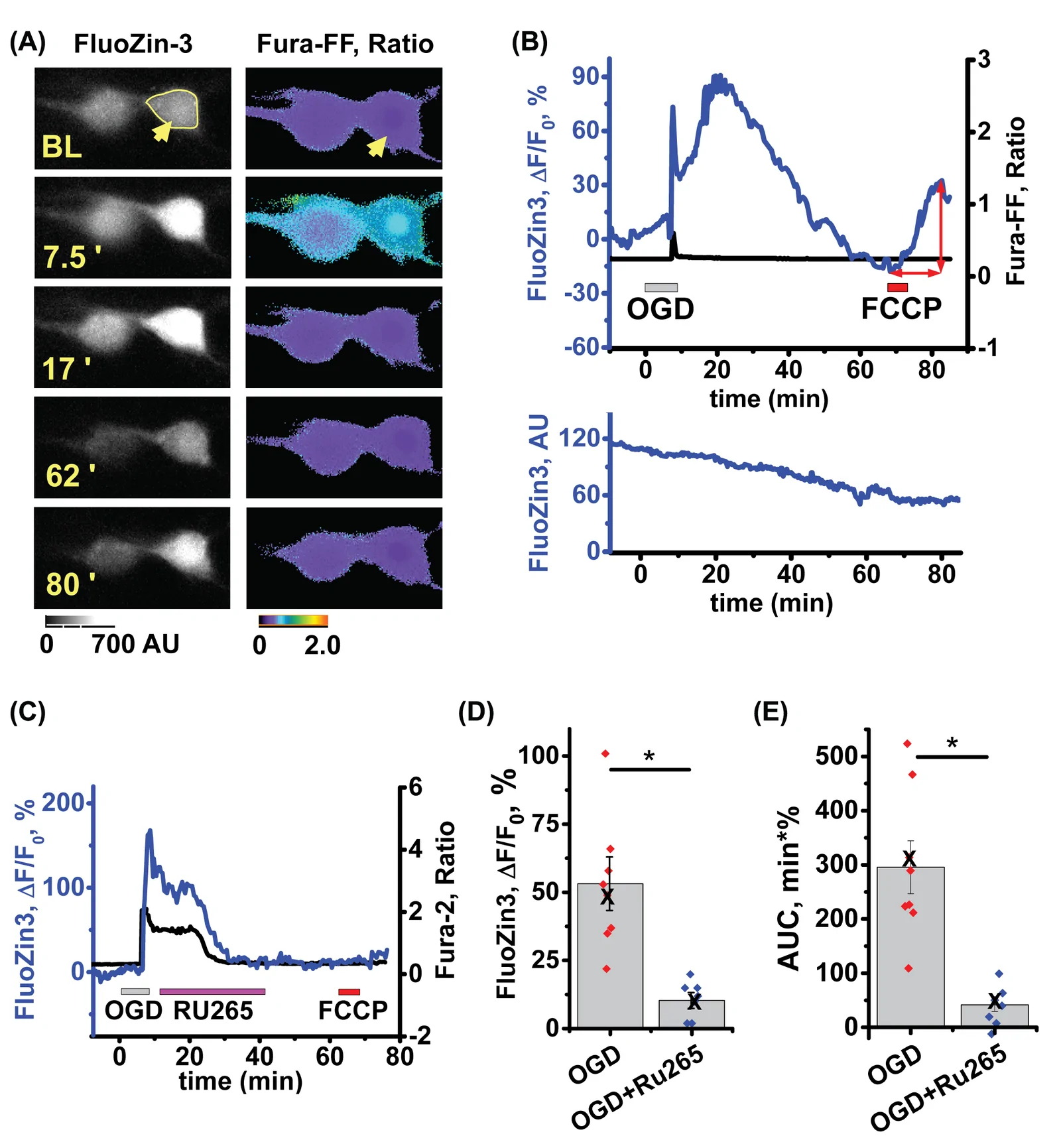
MCU inhibition with Ru265 after OGD withdrawal prevents long-lasting Zn^2+^ accumulation into mitochondria (**A–C**) Assessment of post-ischemic mitochondrial Zn^2+^ accumulation in individual CA1 neurons. Neurons were pipette-loaded with FluoZin-3 and either Fura-FF or Fura-2 and subjected to 8 min OGD. At ∼3 min after OGD withdrawal, Ru265 (5 μM) or placebo treatment was applied for 30 min, and FCCP (2 μM) administered at 1 h post-OGD as indicated. (A) Images show changes in FluoZin-3 fluorescence and in Fura-FF ratio in representative CA1 neuron subjected to OGD followed by FCCP after 1 h. The time marks on images indicate the time elapsed after OGD onset at which each image was acquired. Note that the second neuron visible in the field went out of focus during recording and was therefore excluded from analysis. (**B**) Upper traces show FluoZin-3 fluorescence (Δ*F*/*F*_0_ × 100%, blue) and Fura-FF ratio (black) changes in the CA1 neuron marked with an arrow in (A). The region of interest used for recordings is denoted on the baseline (BL) FluoZin-3 image (A, upper left). The red vertical arrow illustrates the measurement of FCCP-induced FluoZin-3 amplitude increase; additionally, the red vertical and horizontal arrows together demarcate the AUC presented in (E). Lower trace represents background-subtracted FluoZin-3 fluorescent changes in the control CA1 neuron. (C) Traces show FluoZin-3 fluorescence (Δ*F*/*F*_0_ × 100%, blue) and Fura-2 ratio (black) changes in the CA1 neuron subjected to OGD and treated with Ru265 after reperfusion; FCCP was administered 1 h following OGD withdrawal. (**D**) Bar graph shows mean FCCP-induced FluoZin-3 fluorescence increases (±SE); individual symbols represent measurements from single neurons. (**E**) Bar graph show mean AUC (±SE) of FCCP-induced FluoZin-3 fluorescence changes calculated from the onset of FCCP administration over the subsequent 15 min; symbols show individual data points for each neuron. Neurons corresponding to representative traces shown in (B,C) are indicated by an X. *P < 0.01, *t*-test, control: *n* = 8 neurons from 8 slices, 8 mice; Ru265: *n* = 7 neurons from 6 slices, 6 mice.

#### Evaluation of changes in ∆Ψ_m_

To study changes in mitochondrial function we evaluated ∆Ψ_m_. Slices were bath loaded with the ∆Ψ_m_-sensitive indicator, Rhodamine 123 (Rhod123, 26 μM, 30 min, 22–24°C). Rhod123 is positively charged and accumulates in negatively charged mitochondria where its fluorescence is quenched. Upon loss of ∆Ψ_m_ the indicator is released into the cytoplasm causing an increase in fluorescence [26]. FCCP (2 μM) was administered to control slices and 4 h post-OGD to evoke full loss of ∆Ψ_m_ and a consequent increase in Rhod123 fluorescence, to evaluate the level of ∆Ψ_m_ before FCCP application. Rhod123 was excited at 540(25) nm, and emitted fluorescence collected at 605(55) nm. Images were acquired every 15 s, background subtracted, and analyzed. Data are presented as δ*F*/*F*_0_ = (*F* − *F*_0_)/*F*_0_ × 100%. Regions of interest were monitored in the CA1 pyramidal cell layer. For background subtraction we used image of unloaded slices obtained using the same parameters as Rhod123-loaded slices.

#### Assessment of cytoplasmic Ca^2+^ levels using an expressible Ca^2+^ indicator

To evaluate the cytoplasmic Ca^2+^ concentration [Ca^2+^]_i_ in multiple CA1 neurons we used slices from Salsa6f/CaM mice. These mice express a protein-based ratiometric Ca^2+^ indicator in forebrain pyramidal neurons. “Salsa6f” was produced by fusing a protein based Ca^2+^ indicator (GCaMP6f, *K*_d_ ∼375 nM, green fluorescence) and a cell marker (tdTomato, red), permitting ratiometric measurement of cytoplasmic Ca^2+^ rises (as green/red fluorescence ratio). Transgenic mice with Salsa6f targeted to the Rosa26 locus were crossed with CaMKIIalpha-cre T29-1 transgenic mice, yielding expression of “Salsa6f” to forebrain pyramidal neurons. GCaMP6f was excited at 482(20) nm and emitted fluorescence collected at 532(40) nm. TdTomato was excited at 540(25) nm, and emitted fluorescence collected at 605(55) nm. Changes in Ca^2+^ values are presented as the ratio of background subtracted GCaMP6f and tdTomato emission intensities. For background subtraction we used images of control, non-fluorescent slices, as for Rhod123 experiments.

### CA/CPR and Ru265 administration

The presented study utilizes an asphyxial CA + CPR model as previously described [27–29] (see Figure 1B for experiment timeline). Shortly before experiment, rats were anesthetized with 2% isoflurane in a 50% O_2_/50% N_2_ mixture and intubated with a 14-gauge endotracheal tube (Braun Melsungen AG, Melsungen, Germany), connected to a mechanical ventilator and isoflurane vaporizer. The left femoral artery and vein were cannulated for continuous blood pressure monitoring and intravenous drug delivery. Invasive arterial blood pressure was measured continuously using a transducer (CWE Inc., Ardmore, PA). Baseline arterial blood gas (ABG) measurements (Abaxis, Union City, CA) were obtained prior to initiation of asphyxia to ensure that the arterial blood gases were at normal levels before CA.

After surgical preparation, the inhaled gas was switched to 100% O_2_ for 2 min, after which the isoflurane was stopped to wash out anesthesia for 3 min, followed by the inlet disconnection from oxygen to allow room air to be mechanically delivered to the rat. Additionally, neuromuscular blockade was initiated by injection of 1 ml of intravenous Vecuronium (2 mg/kg) with 1 ml of heparinized saline. After 3 min of isoflurane washout, asphyxial CA was induced by turning the ventilator off and clamping the ventilator tubing. CA was defined as systolic blood pressure <30 mm Hg and pulse pressure of 10 mm Hg or less. Duration of asphyxia was 8 min. In the last min of asphyxia, as the ventilator was being reconnected and turned on, epinephrine (0.01 mg/kg) and sodium bicarbonate (1 mmol/kg) were administered intravenously ahead of CPR to stimulate the sympathetic nervous system and manage acidosis, respectively [29]. CPR (manual sternal compressions at 180–240 per min) was performed after 8 min of asphyxia and continued until return of spontaneous circulation (ROSC). Our study used three groups of animals: (1) sham group (control, this group was subjected to all experimental procedure without asphyxia), (2) rats subjected to CA with placebo treatment, and (3) rats subjected to CA followed by intravenous administration of the MCU blocker Ru265 (1 mg/kg) at the time of CPR in order to reach the brain with ROSC (Figure 1B), as we have demonstrated with other drugs administered during the CPR phase [28]. After obtaining post-CA ABG measurements 10 min after ROSC, vessels were decannulated and rats were extubated within the following 1–2 h when weaned off the ventilator.

### Post-CA care and behavioral assessment

After ROSC, animals were monitored and maintained under standard conditions. At ∼4.5-h post-ROSC time point (after the behavioral testing), rats received subcutaneous injections of cefazolin (5 mg/kg), along with a mixture of lactated Ringer’s solution and sterile saline (20 ml/kg) for hydration and infection prevention. Body weight for each rat was recorded after these injections and again at 24 h post-ROSC to calculate overnight weight loss. After injections, rats were returned to the vivarium for recovery and continued monitoring. Neurological assessments using the Neurological Deficit Score (NDS) were conducted at ∼4 and ∼20 h post-ROSC. All assessments were performed by lab members who were blinded to the experimental conditions. The NDS includes an evaluation of arousal, brainstem reflexes, motor and sensory function, behavior, and seizures, following the previously described evaluation criteria [28]. The rats were then perfused at 24 h post-ROSC for brain tissue collection.

### Histochemistry

#### Cardiac perfusion

Twenty-four hours after CPR, animals were anesthetized with an intraperitoneal injection of sodium pentobarbital, and perfused transcardially with 200 ml of phosphate-buffered saline (PBS) for 2 min, followed by 500 ml of buffer containing 0.002% CaCl_2_, 1.6% NaH_2_PO_4_ and 0.4% NaOH (pH 7.3), 4% PFA, and 1% glutaraldehyde over the next 20 min. After perfusion, the brains were postfixed using 2% PFA/2.5% glutaraldehyde at 4°C overnight, and 10 and 20 μm slices were cut using a vibratome (Leica VT1200).

#### Vanadium acid fuchsin staining (VAF)

To assess neuronal injury, 10 μm sections were stained with VAF largely as previously described [30]. Slices were stained with VAF for 1–2 min, washed with PBS, incubated in 0.01% borax solution for 20–30 s, and rinsed in distilled water. Finally, brain slices were cleared by acetate buffer (pH 3.3) for 30 s and rinsed with distilled water. Stained slices were assessed and photographed using light microscopy. Cell counts of injured versus healthy neurons were carried out by an investigator blinded to experimental conditions.

### Antibody labeling and confocal microscopy

To assess mitochondrial morphology, 20 μm slices were stained with primary antibodies against the mitochondrial outer membrane protein TOM20 (1:200) and secondary anti-rabbit fluorescent antibodies (1:200, DyLight™ 488). The images of the hippocampal CA1 zone were taken using an inverted stage Olympus FV3000 confocal laser scanning microscope (Olympus, Japan) with a high−speed resonance scanner, IX3-ZDC2 Z-drift compensator and Olympus 40× silicone oil objective (UPLSAPO40XS, NA = 1.25, WD = 0.3 mm). A 488 nm diode laser was used for excitation and a high-sensitivity cooled GaAsP PMT was used for detection of signals.

An analysis of mitochondrial size and shape was performed blindly using coded images with ImageJ software as previously described [15]. To ensure that we evaluated only pyramidal neuronal mitochondria, only mitochondria in the immediate proximity of easily recognizable pyramidal neuronal nucleii were analyzed. Each single plane image was adjusted manually using brightness/contrast functions of ImageJ software to provide optimal discrimination of mitochondrial edges from background. We selected cells that showed clearly evident mitochondria in the perinuclear regions in a sharp focus. The mitochondria that were aligned with their long axes parallel to the nuclear membrane and round mitochondria in close nuclear proximity were assessed. Measures of long axes, parallel to, and short axes, perpendicular to nuclear membranes were obtained on all clearly demarcated mitochondria adjacent to and surrounding the nuclear circumference. Overlapping mitochondria were not analyzed. Average parameters were calculated for all measured mitochondria per cell.

### Statistics and data analysis

Statistical differences were assessed using two-sample *t*-tests for two-group comparisons and one- or two-way ANOVA for three or more groups of data (Origin 9.1). All comparisons reflect sets of data substantially interleaved in time. Evaluations of mitochondrial and neuronal health and morphology were performed blindly using coded images.

#### *In vitro* experiments

Since our preliminary experiments did not find any differences in investigated parameters between slices of female and male animals, all further analysis included slices from animals of both sexes. We performed 1–2 slice experiments per animal per condition. All comparisons were made based on *n*≥8 slices. For evaluation of neuronal Ca^2+^ levels using Salsa6F/CaM mice, data were collected from single neurons (≥6 neurons per animal).

#### *In vivo* experiments

To analyze neuronal injury (using VAF staining), we evaluated 3–5 sections per animal with 50–100 cells examined per section. All data obtained from *in vivo* experiments were averaged per animal.

#### Behavioral data

Data were analyzed using IBM SPSS Statistics (Version 30.0; IBM Corp., Armonk, NY) and GraphPad Prism (Version 10.5; GraphPad Software Inc., La Jolla, CA). Normality was assessed with the Shapiro–Wilk test. Data for NDS and NDS sub-scores did not meet normality assumptions, thus the comparisons between the groups were done using the Mann–Whitney *U* test. For behavioral data analysis, significance was defined as *P*<0.05.

## Results

### Post-ischemic MCU blockade prevents delayed Zn^2+^ uptake into mitochondria

In order to evaluate the neuroprotective potential of the MCU inhibitor Ru265 [19] after brain ischemia, we first assessed its effect on post-ischemic mitochondrial Zn^2+^ accumulation. Experiments were conducted using the mouse hippocampal slice OGD model of transient ischemia developed in our laboratory [15,16]. CA1 pyramidal neurons were loaded with the membrane-impermeable Zn^2+^-sensitive indicator, FluoZin-3 [24,25], and a Ca^2+^-sensitive ratiometric indicator (Fura-FF or Fura-2) via patch-pipette prior to subjecting the slices to a short (8 min) OGD episode. This technique allows discrimination of Zn^2+^ versus Ca^2+^ signals in the cytoplasm of CA1 neurons [23] confirming the specificity of FluoZin-3 for the detection of cytoplasmic Zn^2+^ dynamics. OGD evoked a sharp rise in cytosolic Zn^2+^ levels, which largely recovered to baseline within 10–30 min following OGD termination and stayed low for the remainder of the 1-h recording period (Figure 2A). To assess whether this Zn^2+^ accumulates into mitochondria, slices were exposed to a high level of the protonophore, FCCP (2 μM), which rapidly depolarizes mitochondria, thereby releasing sequestered Zn^2+^ into the cytosol. Administration of FCCP at 60 min post-OGD resulted in a sharp rise in cytosolic Zn^2+^ levels, indicating substantial Zn^2+^ accumulation within mitochondria (Figure 2A,B,D,E). In contrast, we did not observe any FCCP-evoked increase in intracellular Ca^2+^ levels, indicating an absence of significant mitochondrial Ca^2+^ accumulation at this delayed time point (Figure 2A,B). These data correlate with our previous observations of long-lasting mitochondrial Zn^2+^—but not Ca^2+^—accumulation in CA1 neurons post-OGD [15]. We suggest that after ischemia termination, both Zn^2+^ and Ca^2+^ enter mitochondria via the MCU; however, Ca^2+^ is relatively rapidly extruded by the Na^+^/Ca^2+^ exchanger [31–33], whereas the Zn^2+^ remains sequestered within the mitochondria for a prolonged period.

In a subsequent set of experiments, the MCU antagonist Ru265 [19] was applied for 30 min beginning 3 min after OGD termination. Under these conditions, FCCP application at 60 min post-OGD no longer elicited a cytosolic Zn^2+^ rise, indicating that Ru265 effectively prevented Zn^2+^ entry into mitochondria (Figure 2C–E).

### MCU blockade following OGD attenuates delayed mitochondrial dysfunction

Previous studies have identified Zn^2+^ uptake through the MCU as a potent driver of mitochondrial damage, including persistent ΔΨ_m_ depolarization, ROS generation, swelling, and fragmentation [15,16,34,35]. Thus, to assess the ability of MCU inhibition to protect mitochondria from ischemia/reperfusion induced persistent dysfunction, we evaluated ΔΨ_m_ at 4 h post-OGD. To measure the level of ΔΨ_m_ depolarization, as an index of mitochondrial dysfunction, we employed the fluorescent indicator Rhodamine123 (Rhod123). This positively charged dye accumulates within negatively charged mitochondria, where its fluorescence is quenched, in proportion to ∆Ψ_m_. Application of the protonophore FCCP at high concentration (2 μM) dissipates the mitochondrial proton gradient, inducing complete ∆Ψ_m_ collapse, resulting in Rhod123 release and a corresponding rise in fluorescence (Δ*F*), thereby providing a measure of ∆Ψ_m_ prior to FCCP exposure. Using this approach, we evaluated ∆Ψ_m_ in the CA1 pyramidal cell layer. At 4 hours post-OGD, ΔΨ_m_ was substantially reduced (by approximately 50%), indicative of severe mitochondrial dysfunction. Post-OGD application of Ru265 (5 μM) for 30 min (starting 3 min after OGD termination) markedly attenuated this delayed ΔΨ_m_ loss, indicating improved mitochondrial functional recovery (Figure 3A,B).

**Figure 3:**
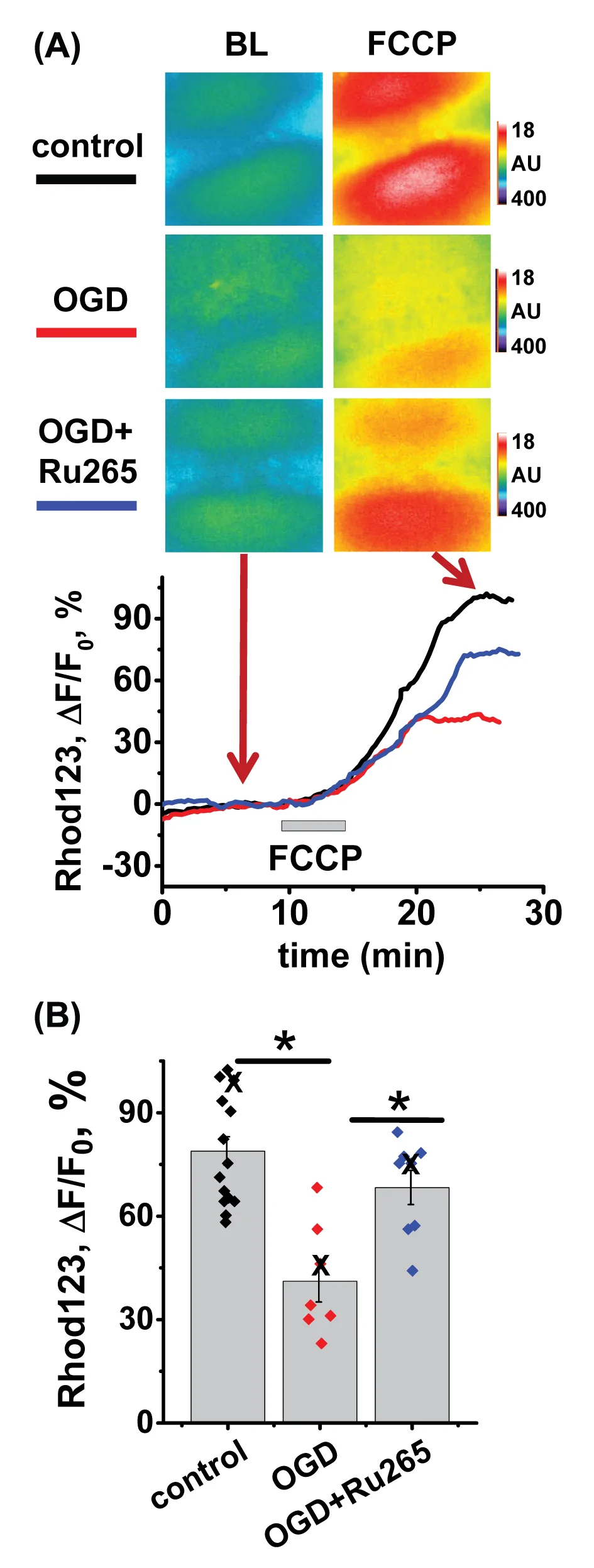
Treatment with Ru265 post-OGD diminishes delayed loss of ∆Ψ_m_ (**A**) Assessment of ∆Ψ_m_. Brain slices were subjected to short OGD (8 min)/reperfusion, followed by placebo treatment or Ru265 (5 μM for 30 min). Next, slices were loaded with Rhod123, and 4 h after OGD termination, FCCP (2 μM) was administered. Upper: Pseudocolor Rhod123 fluorescent images of CA1 area before (baseline, BL) and after FCCP application obtained from representative control slice, and slices subjected to OGD followed by placebo or Ru265 treatment. Lower: Traces represent Rhod123 Δ*F*/*F*_0_ (%) changes in slices shown on the upper images (black—control, red—OGD, blue—OGD + Ru265). Arrows indicate the time points at which fluorescent images were taken. (**B**) Bar graph shows average maximum FCCP-evoked Rhod123 Δ*F*/*F*_0_ × 100% (±SE) increases; symbols represent individual data points for each slice. The symbols depicting Rhod123 increases in slices presented on A are marked with an X. Control: *n*=15 slices from 11 mice, OGD: *n*=7 slices from 7 mice, OGD + Ru265: *n*=8 slices from 7 mice; *P<0.01, ANOVA.

### Delayed MCU blockade attenuates post-ischemic disruption of neuronal Ca^2+^ homeostasis

Disruption of neuronal Ca^2+^ homeostasis is a critical early event in the cascade leading to neurodegeneration after transient ischemia [36–38]. Thus, to further investigate neuroprotective effect of post-ischemic Ru265 treatment, we examined the ability of neurons to maintain physiological intracellular Ca^2+^ concentrations ([Ca^2+^]_i_).

Changes in [Ca^2+^]_i_ were monitored in freshly prepared slices from Salsa6f/CaM transgenic mice, which express the genetically encoded ratiometric Ca^2+^ indicator Salsa6f (GCaMP6f/tdTomato) [39] in forebrain pyramidal neurons, including those in the hippocampal CA1 region. Salsa6f is a fusion protein comprising the Ca^2+^-sensitive green fluorescent indicator GCaMP6f (*K*_d_ ∼375 nM), which exhibits increased green fluorescence upon cytosolic Ca^2+^ elevation, and the Ca^2+^-insensitive red fluorescent protein tdTomato, which provides a stable reference signal enabling ratiometric Ca^2+^ imaging. Notably, at resting [Ca^2+^]_i_ levels, GCaMP6f fluorescence is barely distinguishable from background; therefore, tdTomato was used for cell identification and tracking.

When slices from Salsa6f/CaM were exposed to elevated KCl (90 mM, 3 min), which depolarizes the plasma membrane triggering an activation of voltage gated Ca^2+^ channels (VGCC), a sharp increase in the GCaMP6f/tdTomato fluorescence ratio was observed, consistent with a sharp rise in [Ca^2+^]_i_ that recovered over the subsequent 5–8 min (Figure 4A). Prolonged OGD induces a pronounced terminal [Ca^2+^]_i_ elevation (Figure 4B), similar to our previous observations using synthetic Ca^2+^ indicators [15,23]. Next, we applied transient (8 min) OGD and evaluated delayed changes in [Ca^2+^]_i_. We observed near-control [Ca^2+^]_i_ levels 1-h after OGD termination (GCaMP6f/tdTomato ratio: 0.21 ± 0.01 vs. 0.16 ± 0.01 in control; *P*=0.1). However, at later time points, [Ca^2+^]_i_ progressively increased, reaching a ratio of 0.41 ± 0.01 at 3 h post-OGD (*P*<0.01 vs. baseline), indicative of an impaired ability of neurons to maintain Ca^2+^ homeostasis. In contrast, post-OGD application of Ru265 (5 μM) markedly attenuated this delayed [Ca^2+^]_i_ elevation (0.25 ± 0.02; *P*<0.01 vs. OGD alone) (Figure 4C).

**Figure 4:**
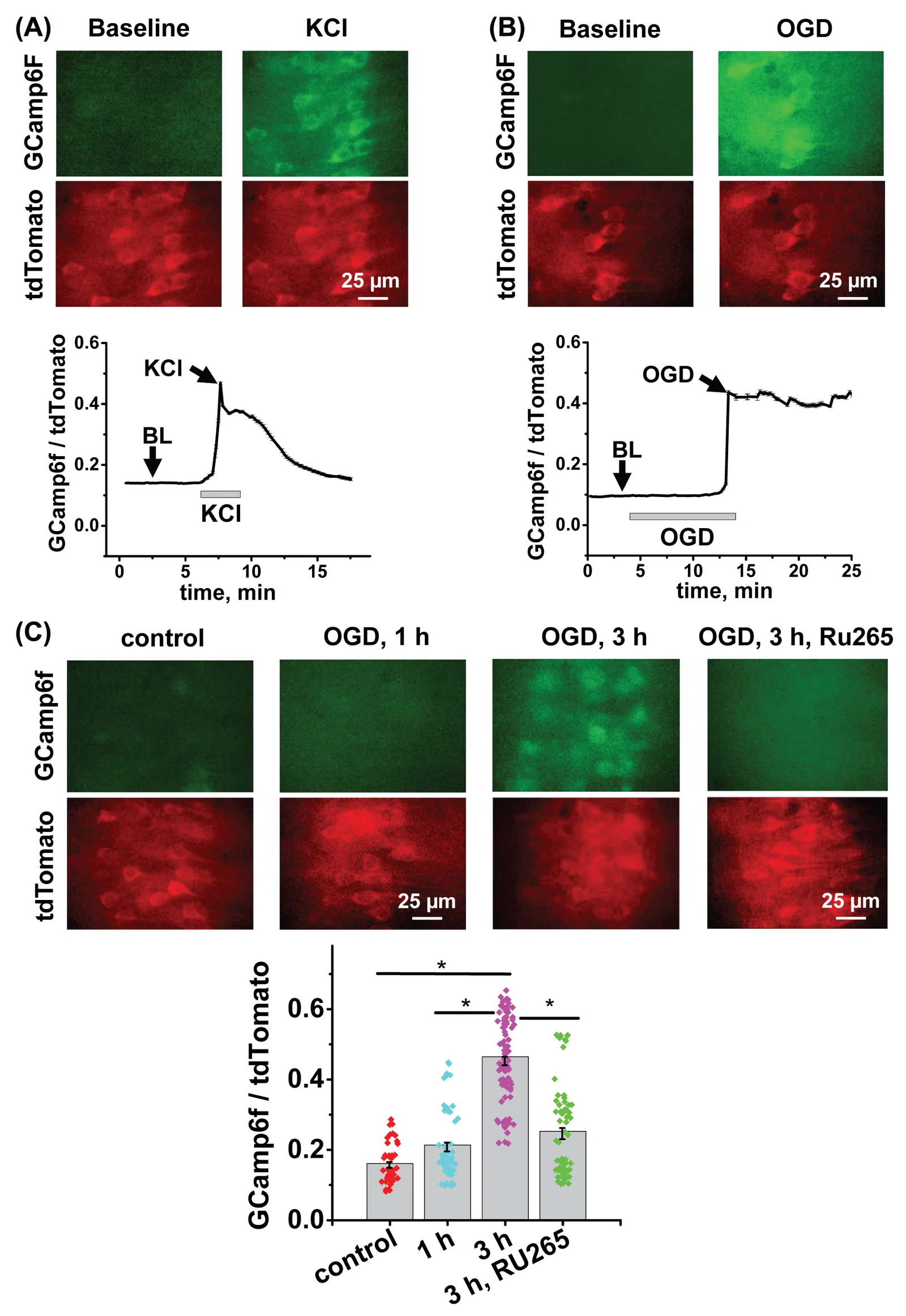
Treatment with Ru265 considerably improves Ca^2+^ homeostasis in CA1 neurons Acute slices were prepared from the brains of Salsa6f/CaM mice. (**A**) Upper—GCamp6f and tdTomato images of CA1 region at baseline and after administration of KCl (90 mM for 3 min) in a representative slice. Lower—Trace illustrates average (±SE) Gcamp6f/tdTomato ratio changes in *n* = 5 neurons. Arrows show time points at which upper images were taken. (**B**) Upper—GCamp6f and tdTomato images of CA1 region before and after subjecting slice to prolonged OGD. Trace shows average changes (±SE) in Gcamp6f/tdTomato ratio in *n* = 4 neurons. (**C**) Brain slices from Salsa6f mice were subjected to OGD (8 min)/reperfusion, and Ru265 (or placebo treatment) was applied 3 min after OGD withdrawal for 30 min. Upper—GCamp6f and tdTomato fluorescent images of CA1 region in representative control untreated slice, 1 and 3 h after OGD alone, and 3 h after OGD + treatment with Ru265, as indicated. Lower—Bar graph represents average (±SE) Gcamp6f/tdTomato ratios, and symbols show Gcamp6f/tdTomato ratios in individual neurons (*n* = 46 neurons from 6 mice in control, *n* = 59 cells from 6 mice at 1 h post-OGD, *n* = 95 neurons from 8 mice at 3 h post-OGD and *n* = 67 neurons from 8 mice at 3 h after OGD + Ru265); *P<0.01, ANOVA.

### Delayed MCU blockade preserves mitochondrial morphology after *in vivo* CA

Subsequent experiments investigated the effects of Ru265 administration following global ischemia *in vivo*. Rats were randomly assigned to one of three experimental conditions: sham treatment, asphyxial CA (8 min) followed by CPR, or CA/CPR with intravenous administration of Ru265 (1 mg/kg) at the time of CPR. The dose employed in the present study is substantially below the 10 mg/kg previously reported to induce seizures in mice [40]. Additionally, no adverse effects were observed in any of the rats treated with 1 mg/kg of Ru265 (*n*=11).

After ∼4 and ∼20 h post-CPR, animals underwent behavioral testing. And ∼24 h after CA, rats were perfused and brains isolated and processed for histological analysis (see Materials and methods).

To assess mitochondrial morphology, 20 μm hippocampal slices were stained with antibody against the mitochondrial outer membrane protein, TOM-20. Confocal images (1000×) were obtained from the CA1 pyramidal cell layer (Figure 5A). Mitochondrial morphological parameters were quantified using ImageJ software, as previously described [15,16]. CA/CPR induced marked mitochondrial fragmentation and swelling, reflected by significant decreases in mitochondrial length, increases in width, and a substantially reduced length-to-width (*L*/*W*) ratio relative to sham-treated animals (Figure 5B–E). Importantly, these morphological alterations were markedly attenuated by Ru265 treatment.

**Figure 5:**
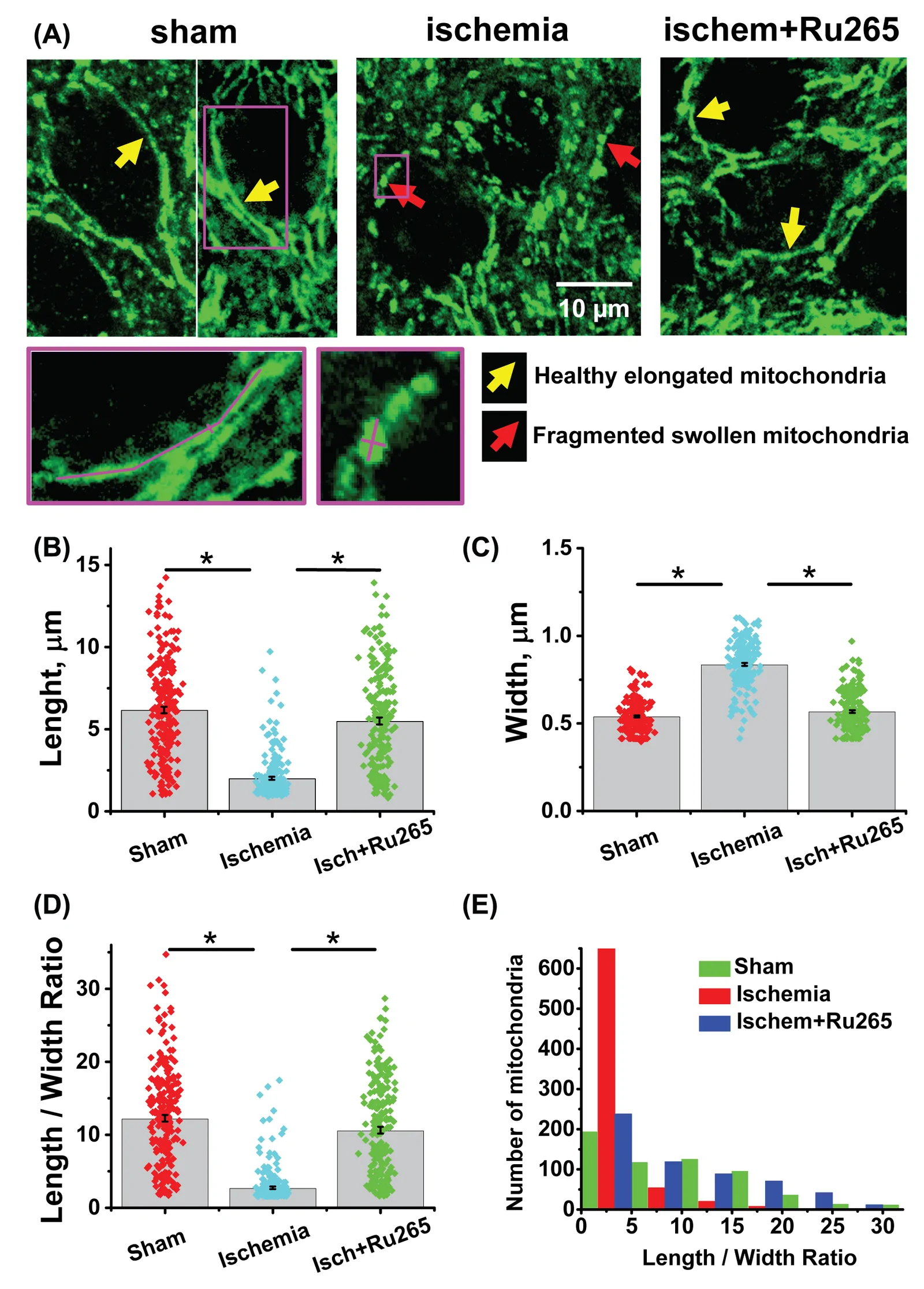
Mitochondrial swelling in CA1 pyramidal neurons 24 h after cardiac arrest is mitigated by MCU blockade with Ru265 Rats were subjected to sham treatment, transient global ischemia induced by CA (8 min), or CA followed by intravenous treatment with Ru265 (1 mg/kg). After 24 h of recovery, rats were euthanized, and brain tissue was collected for histological examination. 20 μm slices were cut with a vibratome and stained with TOM-20 antibody. (**A**) Appearance of mitochondria in CA1 neurons. Representative confocal images show TOM-20 labeled mitochondria. Bottom—Enlarged images of regions indicated on the top panel show representative mitochondria at high magnifications. Inset lines illustrate how measurements of lengths and widths were performed. Note that ischemia caused fragmentation and visible swelling of mitochondria, with a decrease in length and increase in width; and that this change was attenuated by delayed treatment with Ru265. (**B–D**) Quantitative analysis. Bar graphs show mean (±SD) mitochondrial length (B), width (C), and length/width ratio (D). Mitochondrial length and width were averaged for each neuron (>200 cells per animal analyzed). Symbols represent mitochondrial measures averaged per individual neuron. (**E**) Length/width distribution. Bar graphs show *L*/*W* ratio distribution for single mitochondria (*n*>500 for each group) with bin size 5. Data obtained from 3 sham rats, 4 ischemia rats, and 3 ischemia + Ru265 rats; *P<0.01, ANOVA.

### MCU inhibition after CA is neuroprotective

Our previous work demonstrated that brief episodes of global brain ischemia cause considerable injury of CA1 neurons 4 h after CPR [41]. Here, we evaluated the extent of neuronal injury across multiple brain regions at 24 h post-ischemia and assessed the neuroprotective effect of post-ischemic MCU inhibition with Ru265. Neuronal injury was assessed using a modified VAF staining procedure, which selectively labels acidophilic (injured) neurons. Stained sections were examined under light microscopy, and each identifiable neuron was classified as either healthy or injured. In the hippocampus, CA1 pyramidal neurons exhibited severe injury, whereas CA3 pyramidal neurons were considerably less affected, consistent with previously reported patterns of selective vulnerability [5–7]. Importantly, neuronal injury in both CA1 and CA3 was markedly reduced in Ru265-treated animals (Figure 6).

**Figure 6:**
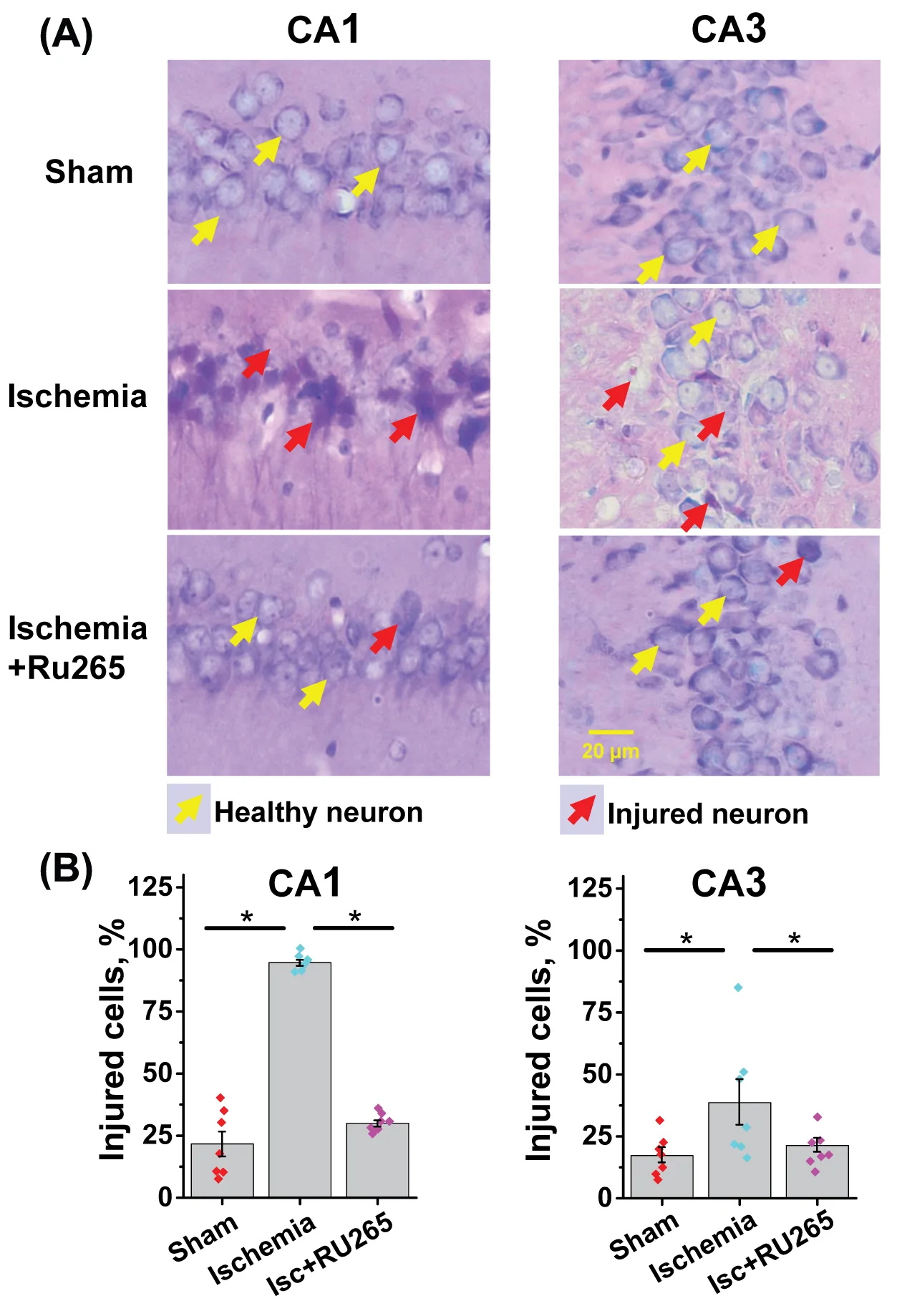
MCU inhibition considerably decreases neuronal injury in CA1 pyramidal neurons after cardiac arrest Rats were subjected to sham treatment, transient global ischemia induced by CA (8 min), or CA followed by intravenous injection of Ru265 (1 mg/kg). After 24-h recovery, rats were euthanized, and brain tissue collected for histological examination. To assess injury, slices were subjected to a modified acid fuchsin labeling procedure (VAF). (**A**) Representative images of VAF-stained CA1 and CA3 regions in brain slices. Note that the majority of neurons appear healthy (yellow arrows) in slices from sham treated animals, but after ischemia most neurons are injured (red arrows) and have VAF labeling in cytoplasm or are swollen with no clear cellular membrane. Note that the injury is considerably less in the CA3 region and also that the injury was largely reversed in both regions, in animals treated with Ru265. (**B**) Quantitative assessment. All neurons were blindly analyzed and rated as healthy or injured, and the percentages of neurons determined to be injured were calculated for each animal. Note that the number of injured cells was far greater in animals subjected to ischemia than after sham treatment, and that Ru265 administration greatly diminished neuronal injury. Bars represent mean (±SE) from 7 rats sham, 7 animals after CA, and 8 rats after CA+Ru265. Symbols show number of injured cells calculated per individual animal; *P<0.01, ANOVA.

In addition to the hippocampus, certain cortical neurons are known to be preferentially vulnerable to ischemic injury [8]. Consistent with this, pyramidal neurons in cortical layers III and V were substantially injured following CA/CPR. Notably, as observed in the hippocampus, Ru265 treatment significantly attenuated cortical neuronal injury (Figure 7).

**Figure 7:**
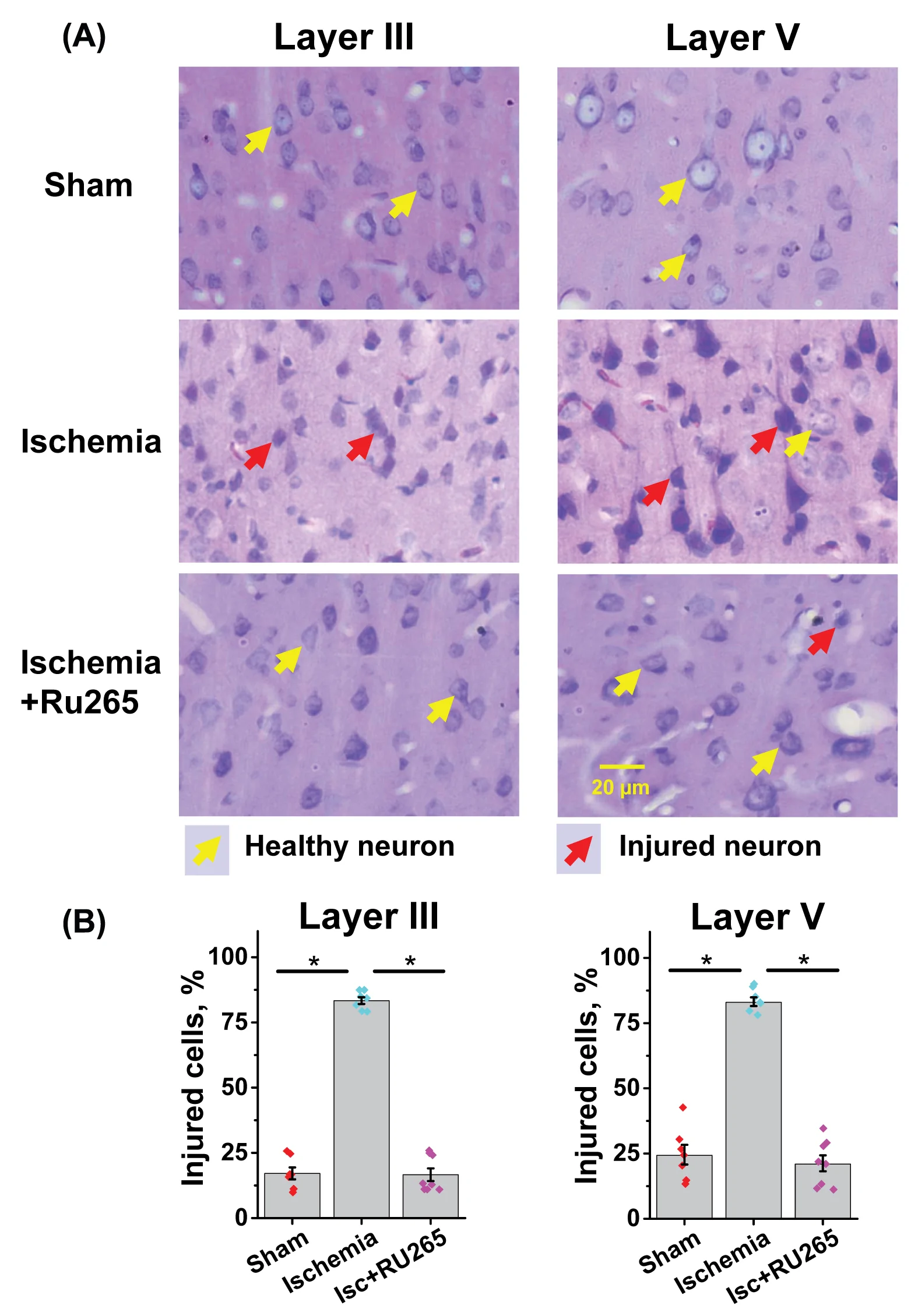
MCU inhibition with Ru265 after CA/CPR considerably decreases neuronal injury in cortical layer III and V neurons (**A**) Representative images of cortical layer III and V. Note that the majority of neurons appear healthy (yellow arrows) in slices from sham treated animals, but after ischemia most neurons are injured (red arrows) and have VAF labeling in cytoplasm. Note that injury was considerably diminished in cortex from Ru265-treated animals. (**B**) Quantitative assessment. All neurons were blindly analyzed and rated as intact or injured, and the percentages of neurons determined to be injured within each animal were calculated. Bars represent mean (±SE) from 7 animals sham, 7 animals after CA, and 8 animals after CA+Ru265. Symbols show numbers of injured cells calculated per individual animal; *P<0.01, ANOVA.

### Delayed MCU blockade improves neurological recovery after CA

To assess neurological function following CA, we employed the Neurologic Deficit Scale (NDS). This rodent behavioral assessment is modeled after the Glasgow Coma Scale used in humans, with scores ranging from 0 (dead) to 80 (neurologically intact) [42,43]. The NDS provides a cumulative score across multiple domains, including arousal, brainstem function, motor and sensory function, and behavioral responses. CA alone produced a substantial reduction in NDS scores relative to sham-treated controls. However, intravenous Ru265 administration after CA markedly improved NDS scores to near-control values at 20 h post-CPR (Figure 8). Note that 4 h after CPR the NDS score was similar in Ru265- and placebo-treated animals (*P*=0.37), reflecting the slow recovery after CA-induced coma.

**Figure 8:**
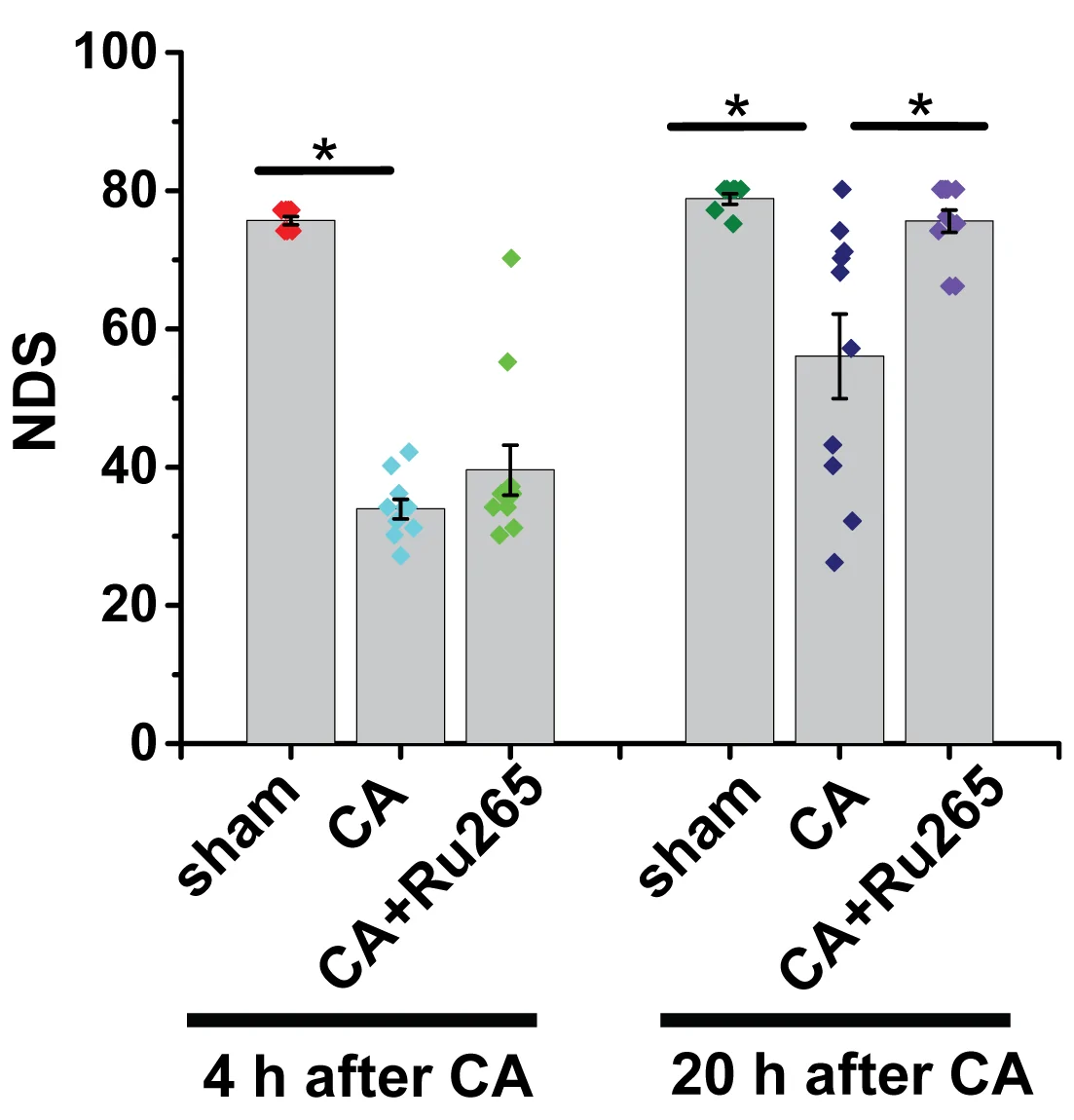
MCU inhibition reverses neurological deficit caused by transient brain ischemia The NDS, a cumulative scores for multiple tests evaluating arousal, brainstem function, motor, and sensory assessment, was used to evaluate the effect of CA and the treatment with Ru265 after CA on animal behavior. Bar graphs represent average NDS in animals 4 and 20 h after CA, as indicated, and symbols show individual data points for each group; *n*=7 sham-treated animals, *n*=10 animals subjected to CA and *n*=11 rats subjected to CA+Ru265, *P<0.05, Mann–Whitney *U* test.

## Discussion

Our previous and current studies have detected progressive, long-lasting Zn^2+^ accumulation in mitochondria of selectively vulnerable CA1 neurons following both *ex vivo* and *in vivo* ischemia [15,41]. This delayed Zn^2+^ accumulation occurs predominantly via the MCU and contributes to ROS overproduction, mitochondrial dysfunction, and subsequent neuronal injury at 4 h post-ischemia [15,16,44]. As the limitations of previously available MCU inhibitors constrained both mechanistic conclusions and translational potential, the present study employed Ru265, a recently developed, highly potent, and relatively selective MCU inhibitor. In the *ex vivo* acute brain slice model, Ru265-mediated MCU blockade substantially decreased mitochondrial dysfunction and restored neuronal Ca^2+^ homeostasis at 3–4 h after OGD. In the *in vivo* rat CA model, intravenous Ru265 administration following CA markedly preserved mitochondrial morphology and considerably reduced neuronal injury in vulnerable hippocampal and cortical regions at 24 h post-CA. Furthermore, MCU inhibition reversed CA-induced behavioral deficits at 20 h post-CPR.

Thus, these findings identify mitochondrial Zn^2+^ accumulation as a viable therapeutic target in the post-ischemic period. The neuroprotective efficacy of Ru265 demonstrated across both *ex vivo* and *in vivo* ischemic models supports its potential as a foundation for future drug development targeting the MCU pathway.

### Understanding mechanisms occurring after restoration of blood flow is key to effective neuroprotection

After transient cerebral ischemia, many vulnerable neurons, particularly CA1 hippocampal pyramidal neurons, initially appear to recover, but subsequently undergo delayed cell death hours to days later. The numerous deleterious cascades driving this neuronal death unfold after reperfusion, at a time when cerebral blood flow has been restored and therapeutic intervention is clinically feasible. Therefore, elucidating the precise sequence of events leading to this delayed neuronal death is essential for developing effective neuroprotective strategies.

Over decades of research, multiple treatments have been proposed and tested, yet most have failed to demonstrate meaningful neuroprotection in humans. This likely reflects the inability of many studies to distinguish between—and selectively target—pathological events occurring after ischemia versus those unfolding during ischemia itself.

One extensively studied mechanism is excitotoxicity, characterized by excessive activation of glutamate receptors (particularly NMDA receptors) leading to rapid neuronal Ca^2+^ overload. Although NMDA receptor blockers demonstrated high efficacy in animal models when administered prior to experimental ischemia, these agents consistently failed to provide neuroprotection in human clinical trials [45,46]. This translational failure is likely attributable to the critical timing of drug administration: during acute ischemic events, massive NMDA-mediated Ca^2+^ influx occurs within minutes, causing neuronal damage well before therapeutic intervention is clinically feasible. Consequently, post-ischemic administration of NMDA receptor blockers likely had minimal impact on the progression of ischemic damage, as the primary excitotoxic insult had already occurred before treatment could be initiated.

Recent advances in reperfusion strategies following stroke opened new possibilities for neuroprotective interventions [47]. Identifying and targeting pathological events that occur specifically after the restoration of blood flow is crucial for the successful development of clinically translatable neuroprotective therapies.

### Mitochondrial Zn^2+^ accumulation through the MCU is a key contributor to ischemic injury

Multiple studies support a critical role of Zn^2+^ in ischemic neuronal injury. Considerably elevated Zn^2+^ levels have been detected in vulnerable neurons after ischemia, including hippocampal CA1 neurons [48,49]. Consistent with this, Zn^2+^ chelation confers neuroprotection in both *in vitro* and *in vivo* ischemia models [23,48,50,51].

Although the brain contains substantial amounts of Zn^2+^, under physiological conditions it is sequestered in distinct Zn^2+^ pools. In neurons there are two major pools: (1) Zn^2+^ sequestered in synaptic vesicles, which is released upon strong synaptic stimulation and can enter neurons through certain Ca^2+^ permeable channels [14,35]; and (2) Zn^2+^ bound to cytoplasmic Zn^2+^-binding proteins (in neurons, primarily metallothionein-III; MT3), from which it can be liberated by oxidative stress and acidosis which occur during ischemia/reperfusion [52–54]. Zn^2+^ release from MT3s appears to contribute substantially to post-ischemic CA1 neuronal injury [15,55,56].

While the mechanisms though which Zn^2+^ causes neuronal damage remain incompletely defined, multiple studies have identified mitochondria as the primary target of Zn^2+^ toxicity. Zn^2+^ enters mitochondria via the MCU and contributes to mitochondrial dysfunction, swelling and ROS generation [55,57–60]. Additionally, Zn^2+^ has been found to induce irreversible inhibition of several mitochondrial enzymes involved in energy production and antioxidant defense, likely contributing to increased mitochondrial ROS production and mPTP induction [61,62]. The mPTP induction, in turn, triggers release of apoptotic mediators including cytochrome *c* and apoptosis inducing factor [58], contributing to the activation of apoptotic injury pathways [63–66]. The ROS overproduction, in addition to damaging all cellular molecules, with particular impairment to mitochondrial DNA [67], triggers inflammatory cascades with immune cell infiltration, further exacerbating injury [68–73].

Our recent studies using *ex vivo* and *in vivo* ischemia models found that after short ischemic episodes Zn^2+^ accumulates in mitochondria via the MCU, where it persists for prolonged periods contributing to ROS overproduction, delayed mitochondrial dysfunction, structural damage, swelling, and fragmentation [15,16,41,44]. While Ca^2+^ also enters mitochondria following ischemia it is likely cleared relatively quickly via the Na^+^/Ca^2+^ exchanger, since we did not observe significant amount of Ca^2+^ in CA1 mitochondria 1 h after ischemia [15]. Importantly, while mitochondrial Zn^2+^ accumulation appears to exert a substantially more potent deleterious effect than cytoplasmic Zn^2+^ [51], cytoplasmic Ca^2+^ accumulation has also been shown to contribute to injury, in part through activation of NADPH oxidase [44,74,75].

The present study demonstrates that MCU inhibition with Ru265 following ischemia prevents mitochondrial Zn^2+^ accumulation, attenuates delayed mitochondrial injury, restores Ca^2+^ homeostasis, reduces neuronal damage, and considerably improves behavioral recovery after CA. Taken together, these findings establish mitochondrial Zn^2+^ accumulation via the MCU as a critical and therapeutically targetable event in the post-ischemic cascade.

### The novel MCU inhibitor Ru265 as a platform for future drug development

Until recently, pharmacological MCU blockade was not clinically achievable, as all available inhibitors were either highly non-selective and therefore unsuitable for human use (Ruthenium Red) [17] or membrane-impermeable and highly susceptible to oxidative degradation (RU360). The recent development of Ru265 has yielded an MCU antagonist with substantially greater selectivity and sufficient potency for use at relatively low doses, as we found that 1 mg/kg produced no overt adverse effects in rats, making it a promising foundation for future translational drug development.

The present study is an early investigation demonstrating that Ru265 represents a promising starting point for drug development for post-ischemic neuroprotective treatment. Future studies should more rigorously evaluate this compound, including assessment of its effectiveness across both sexes, as well as neuroprotective benefits measured at delayed time points—weeks and months post-ischemia. Furthermore, examining its efficacy when administered not only immediately following ischemia but also hours after the ischemic event would be particularly relevant for stroke patients, where timely interventions are often limited. Additionally, the development of improved Ru265 derivatives with reduced side effect profiles would further advance the therapeutic potential.

Ischemic injury is a complex process involving multiple deleterious events that collectively drive delayed neurodegeneration. Mitochondrial Zn^2+^ accumulation and ROS generation [44] appear to represent early initiating events in this pathological cascade, followed by—and likely contributing to—activation of apoptosis-triggering caspases, microglial activation, neutrophil infiltration, and broader immune system involvement, all of which perpetuate and amplify delayed tissue damage [7,68,69]. Ultimately, combined therapeutic strategies targeting distinct components of the downstream injury cascade at specific time points may provide substantial protection against delayed ischemic injury. Early post-ischemic MCU blockade could prove to be a highly effective component of such multimodal treatment strategies.

## Clinical perspectives

- Rationale: Despite being a leading cause of morbidity and mortality in the aging population, brain ischemia lacks effective neuroprotective interventions, largely due to the challenges of pre-reperfusion drug delivery and a limited understanding of the post-reperfusion mechanisms driving delayed neurodegeneration.
- Key findings: Following transient ischemia, zinc ions selectively accumulate within the mitochondria of vulnerable hippocampal CA1 pyramidal neurons, where they persist for prolonged periods and contribute to progressive mitochondrial dysfunction and delayed cellular injury. Notably, post-ischemic administration of Ru265—a selective blocker of the MCU, which serves as the primary route for mitochondrial Zn^2+^ uptake—markedly attenuates delayed neuronal injury and improves neurological outcomes following brain ischemia.
- Clinical significance: These findings offer compelling evidence that administration of MCU antagonists, initiated after the restoration of cerebral blood flow—a clinically relevant therapeutic window—represents a novel and actionable therapeutic strategy for mitigating post-ischemic brain injury.

## Data Availability

Data that support our findings are available from the corresponding author upon reasonable request.

## Open Access

Open access for this article was enabled through an open access agreement between the Biochemical Society/Portland Press and the University of California.

## Abbreviations

ACSF: artificial cerebrospinal fluid
AUC: area under the curve
CA: cardiac arrest
CPR: cardiopulmonary resuscitation
FCCP: carbonyl cyanide-4-(trifluoromethoxy)phenylhydrazone
MCU: mitochondrial Ca^2+^ uniporter
OGD: oxygen–glucose deprivation
Rhod123: Rhodamine 123
ROS: reactive oxygen species
RR: Ruthenium Red
Salsa6f/CaM mice: GCaMP6f/CaMKIIalpha transgenic mice
TPEN: N,N,N’,N’-tetrakis(2-pyridylmethyl)ethylenediamine
